# Effective Treatment Strategies for the Removal of Antibiotic-Resistant Bacteria, Antibiotic-Resistance Genes, and Antibiotic Residues in the Effluent From Wastewater Treatment Plants Receiving Municipal, Hospital, and Domestic Wastewater: Protocol for a Systematic Review

**DOI:** 10.2196/33365

**Published:** 2021-11-26

**Authors:** Mahbub-Ul Alam, Sharika Ferdous, Ayse Ercumen, Audrie Lin, Abul Kamal, Sharmin Khan Luies, Fazle Sharior, Rizwana Khan, Md Ziaur Rahman, Sarker Masud Parvez, Nuhu Amin, Birkneh Tilahun Tadesse, Niharu Akter Moushomi, Rezaul Hasan, Neelam Taneja, Mohammad Aminul Islam, Mahbubur Rahman

**Affiliations:** 1 Environmental Interventions Unit Infectious Disease Division icddr,b (International Centre for Diarrhoeal Disease Research, Bangladesh) Dhaka Bangladesh; 2 North Carolina State University North Carolina, NC United States; 3 University of California Berkeley Berkeley, CA United States; 4 Hawassa University Awassa Ethiopia; 5 Postgraduate Institute of Medical Education and Research Chandigarh India; 6 Washington State University Washington, WA United States

**Keywords:** antimicrobial resistance, antimicrobial-resistant bacteria, antibiotic-resistant bacteria, antimicrobial-resistance genes, antibiotic-resistance genes, antibiotics, antibiotic residues, wastewater treatment plant, effluent, systematic review

## Abstract

**Background:**

The widespread and unrestricted use of antibiotics has led to the emergence and spread of antibiotic-resistant bacteria (ARB), antibiotic-resistance genes (ARGs), and antibiotic residues in the environment. Conventional wastewater treatment plants (WWTPs) are not designed for effective and adequate removal of ARB, ARGs, and antibiotic residues, and therefore, they play an important role in the dissemination of antimicrobial resistance (AMR) in the natural environment.

**Objective:**

We will conduct a systematic review to determine the most effective treatment strategies for the removal of ARB, ARGs, and antibiotic residues from the treated effluent disposed into the environment from WWTPs that receive municipal, hospital, and domestic discharge.

**Methods:**

We will search the MEDLINE, EMBASE, Web of Science, World Health Organization Global Index Medicus, and ProQuest Environmental Science Collection databases for full-text peer-reviewed journal articles published between January 2001 and December 2020. We will select only articles published in the English language. We will include studies that measured (1) the presence, concentration, and removal rate of ARB/ARGs going from WWTP influent to effluent, (2) the presence, concentration, and types of antibiotics in the effluent, and (3) the possible selection of ARB in the effluent after undergoing treatment processes in WWTPs. At least two independent reviewers will extract data and perform risk of bias assessment. An acceptable or narrative synthesis method will be followed to synthesize the data and present descriptive characteristics of the included studies in a tabular form. The study has been approved by the Ethics Review Board at the International Centre for Diarrhoeal Disease Research, Bangladesh (protocol number: PR-20113).

**Results:**

This protocol outlines our proposed methodology for conducting a systematic review. Our results will provide an update to the existing literature by searching additional databases.

**Conclusions:**

Findings from our systematic review will inform the planning of proper treatment methods that can effectively reduce the levels of ARB, ARGs, and residual antibiotics in effluent, thus lowering the risk of the environmental spread of AMR and its further transmission to humans and animals.

**International Registered Report Identifier (IRRID):**

PRR1-10.2196/33365

## Introduction

The role of the environment in contributing to the spread of antimicrobial resistance (AMR) is being recognized on a global scale due to the growing threat it poses to public health. The rapid increase in AMR is rendering even the last generation of antibiotics as useless to treat common infections [1,2]. This has resulted in increased rates of mortality, prolonged hospitalization, and increased health care costs [3]. The widespread and unrestricted use of antibiotics in humans and animals has led to the emergence of antibiotic-resistant bacteria (ARB) and antibiotic-resistance genes (ARGs) by the release of antibiotic residues in the environment through untreated fecal waste [4].

Wastewater serves as one of the largest reservoirs of ARB, ARGs, and antibiotic residues, which have originated from humans, animals, and environments [5,6]. In high-income countries, wastewater resulting from domestic sewage and agricultural runoff is collected at wastewater treatment plants (WWTPs), which are designed to treat wastewater depending on the contaminants in the influent and guidelines for effluent quality [7-9]. Some low-income countries, such as Nigeria, Ethiopia, and South Sudan, do not have WWTPs [10-12]. Low- and middle-income countries (LMICs) usually have WWTPs; however, functionality concerns are common. For example, in India, 54% of WWTPs are operating poorly [12]. LMICs are only able to treat around 28% of the generated wastewater, whereas high-income countries can treat around 70% [13]. WWTPs present in LMICs have often not been designed for the proper removal of ARB, ARGs, and antibiotics [10,14], as evident from ARB and ARGs being detected in wastewater effluent [15,16]. Fecal sludge generated during wastewater treatment is also a major source of ARB, such as extended-spectrum β-lactamase–producing *Escherichia coli* [17]. Discharge of wastewater effluent and fecal sludge containing ARB, ARGs, and antibiotic residues into the environment allows for the dissemination of AMR to other bacteria present in the natural environment. This process is known as horizontal gene transfer, where genetic information is exchanged between neighboring bacteria, leading to increased levels of AMR among populations [15,18].

In addition, wastewater discharged from pharmaceutical industries contains a complex array of contaminants, including antibiotic residues, hormones, toxic substances, and organic compounds, that require novel treatment methods, such as the recent membrane-integrated hybrid technology, which has proven more effective than conventional technologies [19]. However, many pharmaceutical companies around the world continue to conceal the nature and extent of the toxicity of the substances they generate, thus escaping regulations and disposing hazardous waste into the environment [19]. On-site treatment of pharmaceutical waste before release into the sewer system requires technologies that are expensive [10]. In addition to the lack of financial resources and adequate treatment facilities, pharmaceutical companies in LMICs exploit the absence of proper regulatory enforcement and are thus unwilling to employ such methods [20].

Antibiotic residues have been found in greater concentrations in the raw influent of WWTPs in Asian countries, including Japan, China, South Korea, India, Taiwan, Hong Kong, Thailand, Malaysia, Singapore, and Vietnam, compared with European and North American countries [21]. At least nine commonly used antibiotic classes, such as beta-lactams, lincosamides, tetracycline family, vancomycin, chloramphenicol, sulfonamides, fluoroquinolones, macrolides, and trimethoprim, were found in WWTP influent [21]. Antibiotic concentrations found in both influent and effluent from WWTPs reached or exceeded the predicted no-effect concentrations required for resistance selection [21]. A study conducted in India in 2007 found that the effluent from a WWTP receiving waste streams from 90 drug manufacturing companies released a quantity of ciprofloxacin in a single day that was equivalent to the amount sufficient to treat the entire population of Sweden for 5 days [22]. The presence of antibiotics in lethal or sublethal concentrations creates a selective pressure among the bacterial communities present in wastewater, allowing them to acquire resistance through horizontal gene transfer [23].

Conventional wastewater treatment processes combine physical, chemical, and biological treatment levels for the removal of solids and organic matter. The primary sedimentation tank releases effluent for secondary treatment using aerobic biological processes, such as activated sludge processes, trickling filters or biofilters, oxidation ditches, and rotating biological contactors [24]. Afterward, wastewater effluent undergoes tertiary treatment through a disinfection process. One of the most common disinfection methods used at WWTPs is chlorination [25]. These treatment methods have various limitations, including feasibility, efficiency, reliability, environmental impact, sludge production, operation difficulty, pretreatment requirements, and the formation of potentially toxic by-products [26]. High chlorination, treatment costs, long treatment periods, and lack of availability of large areas for WWTP setup are some of the limitations of many chemical-based and biological treatment processes [27]. Inability to remove contaminants, such as ARB, ARGs, and antibiotic residues, poses additional challenges for these conventional processes, proving their effectiveness to be limited in the last 20 years [28].

Therefore, new and additional wastewater treatment technologies have been introduced and have been evaluated for their level of effectiveness in the reduction of ARB/ARGs and antibiotic residues from effluent [29]. Some of these novel processes include membrane filtration systems, UV radiation, ozonation, automatic variable filtration, advanced oxidation processes, and nanotechnology with improved membranes providing efficient energy recovery systems [28,30]. A study comparing the ARB/ARG removal efficiency between chlorination and UV disinfection of wastewater found that consecutive treatment with UV followed by chlorination resulted in a significant reduction in ARGs, in contrast with the application of either method alone [31], whereas, for drinking water treatment, either method led to the effective inactivation of bacterial cells [32]. No single technology alone can obliterate ARB, ARGs, and antibiotic residues in WWTPs [27,33]. To date, many articles have examined the presence or abundance of ARB, ARGs, and antibiotics in the effluent of WWTPs that receive municipal, domestic, hospital, and industrial discharge [33-38]. However, to the best of our knowledge, no study has investigated effective treatment strategies for the removal of ARB, ARGs, and antibiotic residues in WWTPs that simultaneously receive municipal, domestic, and hospital discharge. A knowledge gap also persists in the prevalence of antibiotic residues in wastewater effluents that create selective pressure for bacteria to acquire resistance, which we aim to address through this review.

Therefore, our objective is to compare the efficiency of different new and conventional wastewater treatment methods for reducing the level of ARB/ARGs in effluent and to assess the presence, concentration, and types of antibiotic residues that are released in effluent from WWTPs receiving municipal, domestic, and hospital wastewater. Through the findings of this review, we aim to determine the rate of removal of ARB, ARGs, and antibiotic residues via various wastewater treatment methods, which will eventually allow us to compare the efficiencies of the different methods. The findings will also provide insights into the extent of contamination of ARB, ARGs, and antibiotic residues in water bodies receiving treated wastewater effluent that may pose health risks to humans exposed to those sites. In addition, we aim to determine if wastewater treatment processes in WWTPs promote the selection of ARB despite an effective decrease in the total number of bacteria. Furthermore, a review of the existing level of efficacy of WWTPs will inform future design guidelines for improving wastewater treatment methods in WWTPs that can adequately eliminate ARB, ARGs, and antibiotic residues before environmental release.

## Methods

### Overview

**Figure 1:**
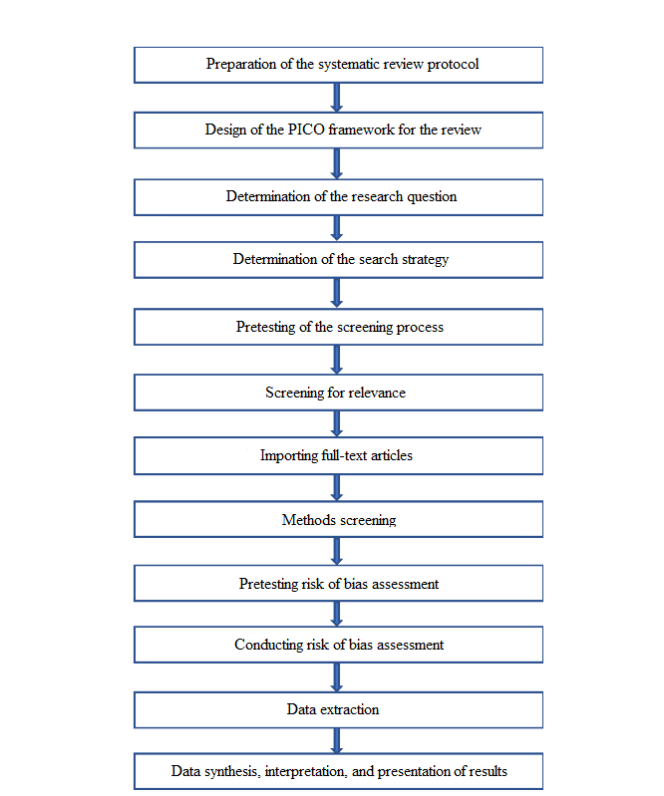
Flow diagram of the steps for conducting the systematic review. PICO: population, intervention, comparison, and outcome.

This systematic review will identify and evaluate various treatment methods in WWTPs that receive municipal, hospital, and domestic discharge with regard to the extent of removal of ARB, ARGs, and antibiotic residues in the effluent released to the environment. We will screen articles for eligibility, perform quality assessment of the studies, extract data, and synthesize the evidence from the published scientific literature (Figure 1).

Textbox 1 outlines the objectives, eligibility criteria, and data sources to be used for the review. The research objectives have been designed following the PICO (population, intervention, comparison, and outcome) framework [39]. Furthermore, we aim to conduct the systematic review by following the PRISMA (Preferred Reporting Items for Systematic Reviews and Meta-Analyses) checklist [40].

**Eligibility protocol for the systematic review.**

**Research Question**

What are the most effective wastewater treatment strategies in wastewater treatment plants (WWTPs) for the removal of antibiotic-resistant bacteria (ARB), antibiotic-resistance genes (ARGs), and antibiotic residues in the resulting effluent?

**Objectives**

- To determine the removal rate of ARB/ARGs in WWTPs that receive municipal, hospital, and domestic wastewater and compare between conventional treatment strategies and new/alternative treatment strategies (eg, presence or absence of disinfection process)

- To assess the presence, concentration, and types of antibiotics in the effluent of WWTPs

- To determine if wastewater treatment processes in WWTPs promote the selection of ARB despite an effective decrease in the total number of bacteria

**Search Strategy**

***Inclusion Criteria***

- Relation of various WWTP treatment methods with antimicrobial resistance

- Estimation of the rate of removal of ARB/ARGs

- Presence and concentration of ARB/ARGs in the influent and effluent from WWTPs that receive municipal, hospital, and domestic discharge

- Presence and concentration of antibiotic residues

- Types of antibiotic residues in the effluent from WWTPs that receive municipal, hospital, and domestic discharge

- Full-text article from a peer-reviewed journal, and other grey materials

- English language

***Exclusion Criteria***

- No inclusion of wastewater treatment methods

- No assessment of ARB or ARGs in the influent and effluent from WWTPs that receive municipal, hospital, and domestic discharge

- No assessment of the presence/concentration of antibiotics in the effluent of WWTPs

- Involvement of WWTPs that receive wastewater from agricultural or industrial/commercial origin, discharge from animal farms or slaughter houses, and only municipal, domestic, or hospital wastewater

- No mention of the types of wastewater that were received by the WWTP

- Collection of pretreated influent samples

- Sample collection during a high epidemic situation

- Pilot/lab-scale studies, books, reviews, guidelines, and survey studies

***Time Frame***

January 2001 to December 2020

**Data Sources**

***Peer-reviewed Articles***

Ovid MEDLINE, Ovid EMBASE, Web of Science, World Health Organization Global Index Medicus, and ProQuest Environmental Science Collection

***Grey Literature***

Google Scholar and Trove

### Population

The population of interest will not be specific to any groups, categories, or locations. Studies that assess the presence and/or abundance of ARB, ARGs, and antibiotic residues in treated WWTP effluent in any population will be included.

### Interventions

Interventions will include the wastewater treatment methods employed at WWTPs that receive municipal, hospital, and domestic wastewater, with respect to the removal of ARB/ARGs and antibiotic residues from the effluent that is discharged into receiving water bodies from the treatment plant.

### Comparison

We will compare conventional and new wastewater treatment strategies to assess the efficiency of different wastewater treatment methods in reducing the levels of ARB, ARGs, and antibiotic residues from effluent. We consider conventional treatment methods as a combination of processes, including physical, chemical, and biological methods for removing solids and organic matter, and new/additional treatment methods as those that provide integrational alternatives for the treatment of wastewater for reducing contaminants.

### Outcomes

The main outcomes to be explored in this systematic review are as follows: (1) The removal rate of ARB/ARGs from WWTPs that receive 3 types of wastewater, and comparison between conventional treatment strategies and new/additional treatment strategies (eg, presence or absence of disinfection processes); (2) The presence, concentration, and types of antibiotic residues in the effluent; and (3) Whether wastewater treatment processes in WWTPs promote the selection of ARB despite an effective decrease in the total number of bacteria.

### Sources of Information

The research team will search several scientific electronic databases for peer-reviewed articles and grey literature to identify published articles according to the search terms for the review.

Databases will include Ovid MEDLINE, Ovid EMBASE, Web of Science, World Health Organization Global Index Medicus (WHO GIM), and ProQuest Environmental Science Collection. Searches for grey literature will be conducted through Google Scholar and Trove. The reference lists of all the included studies will also be searched manually by the researchers to identify any relevant articles for inclusion in the review and for improving the comprehensiveness of the search. Since the review will be based on samples from influent and effluent generated from WWTPs to determine the removal rate of ARB, ARGs, and antibiotic residues, which will help us to determine the treatment methodologies that have a comparative advantage, we will not include any existing review articles on the relevant topic.

Studies that have been published or accepted for publication will be included since limited data from conference proceedings may not allow an in-depth assessment of the studies [41,42] and will be limited to those that have been published within the years 2001-2020. The time frame limitation has been added since studies related to AMR published prior to 2001 did not have an environmental focus. The review will also be limited to English articles that are available in international databases and on websites. However, a certain level of bias may be introduced due to the language restriction, although few studies claim that excluding non-English literature does not have a large effect on systematic reviews and meta-analyses [43,44].

### Search Strategy

The search strategy used for the Ovid MEDLINE database has been shown in Table 1. For the other databases, the search strategy will be adjusted according to the instructions of each database.

**Table 1:** Search strategy used for the MEDLINE database.

Number	Search^a^	Results, n
1	Antibiotic residue$.mp.	1052
2	Antibiotic resistan$ bacteria.mp.	3338
3	antibiotic resistance gene$.mp.	5460
4	antimicrobial resistan$ organism$.mp.	161
5	antimicrobial resistan$ pathogen$.mp.	267
6	ARB selection.mp.	2
7	Anti-Microbial Agent$.mp.	399
8	Wastewater treat$.mp.	23,724
9	Graywater treat$.mp.	9
10	Greywater treat$.mp.	92
11	wastewater treat$ plant$.mp.	10,628
12	wastewater treatment method$.mp.	105
13	conventional wastewater treatment process$.mp.	69
14	new wastewater treatment process$.mp.	11
15	effluent.mp.	29,387
16	influent.mp.	7430
17	Antimicrobial resistance gene$.mp.	1566
18	antimicrobial resistan$.mp.	25,483
19	antibiotic resistan$.mp.	45,671
20	plasmid$.mp.	184,995
21	Sewage treatment.mp.	3861
22	1 or 2 or 3 or 4 or 5 or 6 or 7 or 17 or 18 or 19 or 20	245,158
23	8 or 9 or 10 or 11 or 12 or 13 or 14 or 15 or 16 or 21	52,791
24	22 and 23	1397
25	limit 24 to yr=“2001 -Current”	1324
26	limit 25 to english language	1296

^a^In the search, mp=title, abstract, original title, name of substance word, subject heading word, floating sub-heading word, keyword heading word, organism supplementary concept word, protocol supplementary concept word, rare disease supplementary concept word, unique identifier, and synonyms.

### Data Management

The articles retrieved from database searching will be imported to EndNote X9 to identify and remove duplicates. Remaining entries will be evaluated using the inclusion and exclusion criteria. The list of retrieved full-text articles will be exported from EndNote to the web-based application Rayyan QCRI, which will be used for carrying out the screening process [45]. Following the PRISMA guidelines, we will select articles in 4 phases following the flow of the diagram as follows: (1) identification, (2) screening, (3) eligibility, and (4) inclusion [46].

### Study Selection

During the study selection process, titles and abstracts of the articles will be screened to shortlist them according to relevance to the objectives under the research question. Two reviewers will screen titles and abstracts independently. The decision for including or excluding articles will be finalized by the 2 reviewers, after reaching a common level of understanding for each article, and a third reviewer will provide approval and resolve disagreements, should any arise. The shortlisted articles will be screened for eligibility in order to determine if they fulfill the objectives of the review, and critical appraisal will be conducted subsequently.

We will use a standardized set of questions that have been adapted from previous studies [42,47,48] for selecting articles to include or exclude for the systematic review. The questions for screening studies for relevance are as follows: (1) Does the abstract refer to primary research published in a peer-reviewed journal or as grey literature (eg, thesis)? (2) Does the study assess the presence, abundance, or concentration of ARB, ARGs, or antibiotic residues in the influent and effluent of WWTPs? (3) Does the study report the removal rate of ARB, ARGs, or antibiotic residues going from influent to effluent? (4) Were the samples collected from the effluent of WWTPs that received municipal, domestic, and hospital discharge?

Articles for which the answer is “Yes” to any of the questions from 1 through 3 and “Yes” for question 4 will be shortlisted from the relevance screening phase for full-text review. Articles for which the answer is “No” for question 4 will be excluded and will not be considered for additional review. Literature for which the answer to the first 3 questions cannot be determined through screening of the abstracts and titles will be considered for further full-text screening. These articles will be classified as “maybe” in Rayyan QCRI software for further discussion among the reviewers in order to reach a mutual decision regarding inclusion. After finalizing the list of articles from the relevance screening phase, we will screen the methods of the included studies against the objectives of the review for data extraction.

### Data Extraction

To increase the reliability of the process, data extraction will be carried out by at least two reviewers who will collaboratively work on the selected articles. Findings extracted will be inputted into a standardized data extraction form on MS Excel spreadsheet (Microsoft Corp) that will be piloted before use. The form will include key points, findings, and a summary of the included studies based on the review objectives. The extracted data will be grouped into the following categories: (1) characteristics of the study (location, study design, and sample size), (2) type of source (location and characteristics of WWTPs), (3) type of sample (quality of samples, ARB or ARGs analyzed, and effluent or influent analyzed), and (4) comparators (types of treatment methods analyzed, and the removal rate, presence, and concentration of ARB/ARGs/antibiotics for each method).

### Quality Assessment

We will perform risk of bias assessment at the study design level through adapting the previously published tool by Williams-Nguyen et al [47]. The risk of bias in each domain will be categorized as “low,” “high,” and “unclear” according to the Cochrane Collaboration Risk of Bias Tool [49]. Two reviewers will independently assess the quality of the included studies. Before the full assessment, the risk of bias tool (Table 2) will be pretested by both reviewers on several included papers to improve interpretation agreement and ensure consistency of data entry. Discrepancies will be resolved by discussion, and a third reviewer will be consulted if necessary.

**Table 2:** Risk of bias tool.

Bias domain	Assessment question	Criteria
Sample selection bias	Were sample locations and sampling methods implementing such that sampling did not introduce systematic differences depending on the value of the exposure variable for each sample (in the case of continuous exposure data) or between the comparison groups (in the case of categorical exposure measures)?	Criteria for the judgement of “Yes” (low risk): Method for determining the sampling locations is identical and independent of exposure status (ie, sample taken from the influent and final effluent of the WWTP^a^) The WWTP receives only municipal, hospital, and domestic wastewater regardless of differences in treatment methods or treatment stages (primary, secondary, or tertiary) Influent wastewater does not have any form of pretreatment before being discharged in the WWTP The time between sampling at all sites is sufficiently close to render the outcomes measured at these sites comparable for the sample type in question Collection of 24-h composite samples The authors describe the frequency of sampling (daily, weekly, monthly, etc) at each site The authors describe the volume of collected samples from the influent and effluent of the WWTP Criteria for the judgement of “No” (high risk): Sampling locations are selected differently (eg, samples taken from the effluent of the grit chamber, aeration tank, and secondary clarifier) The WWTP does not receive municipal, hospital, and domestic wastewater regardless of differences in treatment methods or treatment stages (primary, secondary, or tertiary) Influent wastewaters have some form of pretreatment before being discharged in the WWTP Time between sampling at all sites is not sufficiently close Collection of grab samples Collection of grab samples The authors do not describe the frequency of sampling (daily, weekly, monthly, etc) at each site The authors do not describe the volume of collected samples Risk of bias will be considered “unclear” if there is not enough information to judge sample selection bias criteria as either “yes” or “no.” For example, if methods for determining sampling locations are not described in enough detail.
Information bias	Were outcome ascertainment methods (ie, methods for antibiotic-resistance gene, antibiotic-resistant bacteria, and antibiotic or bacterial measurements) conducted in a way that ensures the same accuracy regardless of wastewater sample type?	Criteria for the judgement of “Yes” (low risk): Identical microbiological methods are applied to all samples (ie, influent and effluent samples) for ARB^b^, ARG^c^, and antibiotic detection (eg, culture, polymerase chain reaction, genotyping, phenotypic tests, mass spectrometry, and high-performance liquid chromatography). Controlling for different laboratory factors (eg, laboratory type, technician, testing date, and instrument used) Criteria for the judgement of “No” (high risk): Application of different methods depending on the comparison group No adjustment strategy for different laboratory methods Risk of bias will be considered “unclear” if there is not enough information to judge information bias criteria as either “yes” or “no.” For example, if methods for analyses are not explained sufficiently to reach a judgement.
Confounding	Were adequate methods to control for potential confounding employed?	Criteria for the judgement of “Yes” (low risk): Restriction of the sample population (eg, samples are not collected on a rainy day and instead collected on a dry day) Samples are collected in different seasons (eg, winter and summer) Analytical confounding control (eg, stratification, regression adjustment, and test samples are stored correctly) Criteria for the judgement of “No” (high risk): The sample population is not restricted (eg, samples are collected on a rainy day) Lack of any confounding control despite being likely (eg, samples are not collected in different seasons [winter and summer] and no consideration of water salinity) Inappropriate method of confounding control (eg, test samples are not stored correctly) Controlling for confounding is correctly applied for some potential confounders, but not for all Risk of bias will be considered “unclear” if there is not enough information to judge information bias criteria as either “yes” or “no.” For example, if methods to control for confounding are mentioned but the implementation is not explained sufficiently at length to reach a judgement.

^a^WWTP: wastewater treatment plant.

^b^ARB: antibiotic-resistant bacteria.

^c^ARG: antibiotic-resistance gene.

### Evidence Synthesis

The reviewers will decide on the aspects of the study for data synthesis by considering accuracy, limitations, and the approach used to assess the effectiveness of the wastewater treatment methods. An acceptable or narrative synthesis method will be followed to synthesize data based on the objectives of the research question, which will be presented in a tabular form. Descriptive characteristics of the study, including study design, location, year, sample type, and quality, as well as variables analyzed in the study, will be provided. Outcomes of the study will be synthesized, which will allow for relevant comparison between treatment methods in WWTPs. Interpretation of the removal rate of the treatment processes for ARB/ARGs and antibiotic residues will be finalized for data synthesis depending on the outcomes in the included studies found after data extraction. We will also present the quality assessment of the included studies and mark the risks as “low,” “high,” and “unclear” in a tabular form. Heterogeneity in the included studies will be considered, and based on the type of findings an additional meta-analysis may be presented separately if sufficient high-quality homogenous studies are found that would allow findings to be pooled for a fixed- or random-effects meta-analysis.

### Ethics Approval

The study was approved by the Ethics Review Board at the International Centre for Diarrhoeal Disease Research, Bangladesh (Protocol Number: PR-20113).

## Results

We will start the review on December 1, 2021, and it will be completed by June 30, 2022. Our systematic review results will provide an update to the existing literature by searching on additional databases. Findings from our study will inform the planning of proper treatment methods that can effectively reduce the levels of ARB, ARGs, and residual antibiotics in effluent, thus lowering the risk of environmental spread of AMR and its further transmission to humans and animals.

## Discussion

The protocol outlines our methods for a systematic review of the published scientific literature to determine the most effective treatment strategies in WWTPs that do not receive industrial wastewater for the removal of ARB, ARGs, and antibiotic residues from effluent. The provided flow diagram in Figure 1 will be followed as a guide for the review process for searching the literature using the specified keywords, screening phases of the shortlisted literature following the standardized questionnaire, conducting quality assessment of the studies, and finally conducting data extraction after retrieval of full-text articles for evidence synthesis.

A systematic review published in 2018 [50] on the role of WWTPs and agricultural facilities in the dissemination of ARB and ARGs in the natural environment explored outcomes that are closely related to this study. However, we aim to provide an update to the existing literature by searching other databases, such as Ovid MEDLINE, Ovid EMBASE, Web of Science, WHO GIM, and ProQuest Environmental Science Collection. This initiative will enrich the current level of understanding of the impact of ARB and ARGs focusing on effluents from WWTPs that receive municipal, hospital, and domestic wastewater.

## Appendix

Multimedia Appendix 1 Peer-review report by the International Centre for Diarrhoeal Disease Research, Bangladesh.

## Abbreviations

AMR: antimicrobial resistance
ARB: antibiotic-resistant bacteria
ARG: antibiotic-resistance gene
LMIC: low- and middle-income country
PRISMA: Preferred Reporting Items for Systematic Reviews and Meta-Analyses
WHO GIM: World Health Organization Global Index Medicus
WWTP: wastewater treatment plant
