# Supplementary material for: Effective Treatment Strategies for the Removal of Antibiotic-Resistant Bacteria, Antibiotic-Resistance Genes, and Antibiotic Residues in the Effluent From Wastewater Treatment Plants Receiving Municipal, Hospital, and Domestic Wastewater: Protocol for a Systematic Review
Source: JMIR Res Protoc. 2021 Nov 26;10(11):e33365. doi: 10.2196/33365 (PMC8665387; doi:10.2196/33365)
Supplement: Multimedia Appendix 1 [file resprot_v10i11e33365_app1.pdf]

Memorandum

15 November 2020

To: Dr Md Mahbubur Rahman
Principal Investigator of research protocol # PR-20113
Infectious Diseases Division (IDD)

From: Dr Shafiqul Alam Sarker, MD, PhD, FRCP
Chairperson
Research Review Committee (RRC)

Subject: Research protocol # PR-20113

Thank you for your memo dated 01 November 2020 submitting your research protocol # PR-20113 titled “Assessing the association of Antimicrobial Resistance to child growth and the environmental impacts of the spread of resistance through wastewaters and agricultural products: A secondary data analysis” for review and approval through expedited review process. Your proposal was referred to a reviewer for review and comment. The reviewer made the following comments:

a. Is this project hypothesis driven such as “here is an association between reported antibiotic use and diarrheal prevalence among children aged <3 years; and reported antibiotic use and child linear growth”- please explain**.**

b. Study time line mentioned June 2020 to December 2020; As per personnel justification 7 more months are required to complete the study, please reschedule the study time line.

c. Sample size: Investigators will use Environmental Enteropathy (EE) data set which is a subset of the WASH-B study; the sample size for the analysis has been finalized to include 1530 participants and will compare between with vs. without reported antibiotic use. However, investigators did not use any sample size calculation, and power of the study.

d. Ethical Assurance for Protection of Human rights: This study will not involve collecting primary data; therefore, they will not use any informed consent form for the study at any time. However, confidentiality of information will be strictly maintained and access of the data will be restricted within the research team members. But, researchers can provide the previous approved consent form in this study.

Please modify the protocol addressing the above observation and submit its revised version for consideration of the Chairperson.

Thank you.

Cc: Senior Director, IDD

Research Review Committee, IRB Secretariat, Research Administration, CMS
International Centre for Diarrhoeal Disease Research, Bangladesh
68, Shaheed Tajuddin Ahmed Sarani, Mohakhali, Dhaka 1212, Bangladesh
Mail: icddr,b, GPO Box 128, Dhaka 1000, Bangladesh
Phone: 880-2-9827084, Web: http://www.icddrb.org
